# Two case reports of renal-splenic disease presenting as renal tumors or metastases, with a literature review

**DOI:** 10.3389/fonc.2025.1551601

**Published:** 2025-08-27

**Authors:** Lijing Xu, Jialin Wang, Guangxi Sun, Hao Zeng

**Affiliations:** ^1^ Department of Urology, West China Xiamen Hospital, Sichuan University, Xiamen, Fujian, China; ^2^ Department of Urology, West China Hospital, Sichuan University, Chengdu, Sichuan, China

**Keywords:** renal splenosis, diagnostic imaging, surgery, metastatic cancer, RCC (renal cell carcinoma)

## Abstract

This article provides a comprehensive review of studies and case analyses on ectopic splenic tissue, with a particular focus on renosplenic disease. Ectopic splenic tissue refers to the abnormal non-physiological localization of spleen tissue, commonly resulting from splenic tissue implantation or hematogenous metastasis following splenectomy. Renosplenic disease is rare and often misdiagnosed as a renal tumor or tumor recurrence, which can lead to unnecessary surgical interventions. By discussing two cases of postoperative renosplenic disease in detail and combining them with a literature review, this article explores the pathogenesis, clinical presentation, imaging characteristics, and diagnostic methods of the condition. Analysis of 39 previously reported cases of nephrosplenopathy revealed that it predominantly affects male patients, typically occurs on the left side, and is often associated with a history of splenectomy, with lesions identified on average 20 years post-splenectomy. The clinical manifestations of nephroplenic disease are nonspecific and are mostly incidental findings during imaging examinations. Hybrid SPECT/CT and SPIO-enhanced MRI are considered the gold standards for diagnosing ectopic splenic tissue. However, the majority of cases are still confirmed through needle biopsy or surgical resection. While surgical diagnosis allows for lesion removal, it also carries risks of postoperative complications, such as intestinal fistula, as reported in one of the cases in this study. Research indicates that ectopic splenic tissue is generally benign but can cause symptoms by compressing adjacent structures. For asymptomatic patients, conservative management or active surveillance is a viable approach. However, in cases of large lesions, the decision between conservative treatment and surgical intervention should be carefully weighed. By summarizing 48 years of nephroplenic disease case data, this article aims to provide a clinical reference for the diagnosis and management of the condition. It emphasizes the critical role of imaging examinations and the potential for conservative treatment, aiming to reduce surgical risks and recovery times while improving diagnostic accuracy, treatment outcomes, and patients’ quality of life.

## Introduction

Ectopic splenic tissue, also known as accessory spleen or splenic heterotopia, is the presence of splenic tissue outside its normal anatomical location. It is characterized by nonphysiological displacement of the spleen and can be classified as either congenital or acquired. Acquired splenic heterotopia, also called splenic disease, is typically caused by spleen injury in which the splenic tissue is locally implanted or hematogenously disseminated to other body parts, and in such regions, it is often misdiagnosed as a tumor (1). Ectopic splenic tissue is uncommon in clinical practice and is usually asymptomatic, often discovered incidentally. Reports frequently describe ectopic splenic tissue in the liver, pancreas, gastric fundus, or abdominal cavity (2). Moreover, ectopic splenic tissue located in the kidney or renal fossa is easily mistaken for renal or recurrent tumor, presenting a significant challenge for urologists. This arises from the difficulty in distinguishing ectopic splenic tissue from renal cell carcinoma or other benign renal tumors (3). Accurate characterization of such tissue masses can help avoid unnecessary partial or total nephrectomy, including its associated risks and complications, in asymptomatic patients. Herein, we report on two patients with a history of tumor and splenectomy in whom incidental findings of tumor recurrence led to reoperation, ultimately resulting in a diagnosis of splenic disease.

## Case history

### Case 1

**Figure 1:**
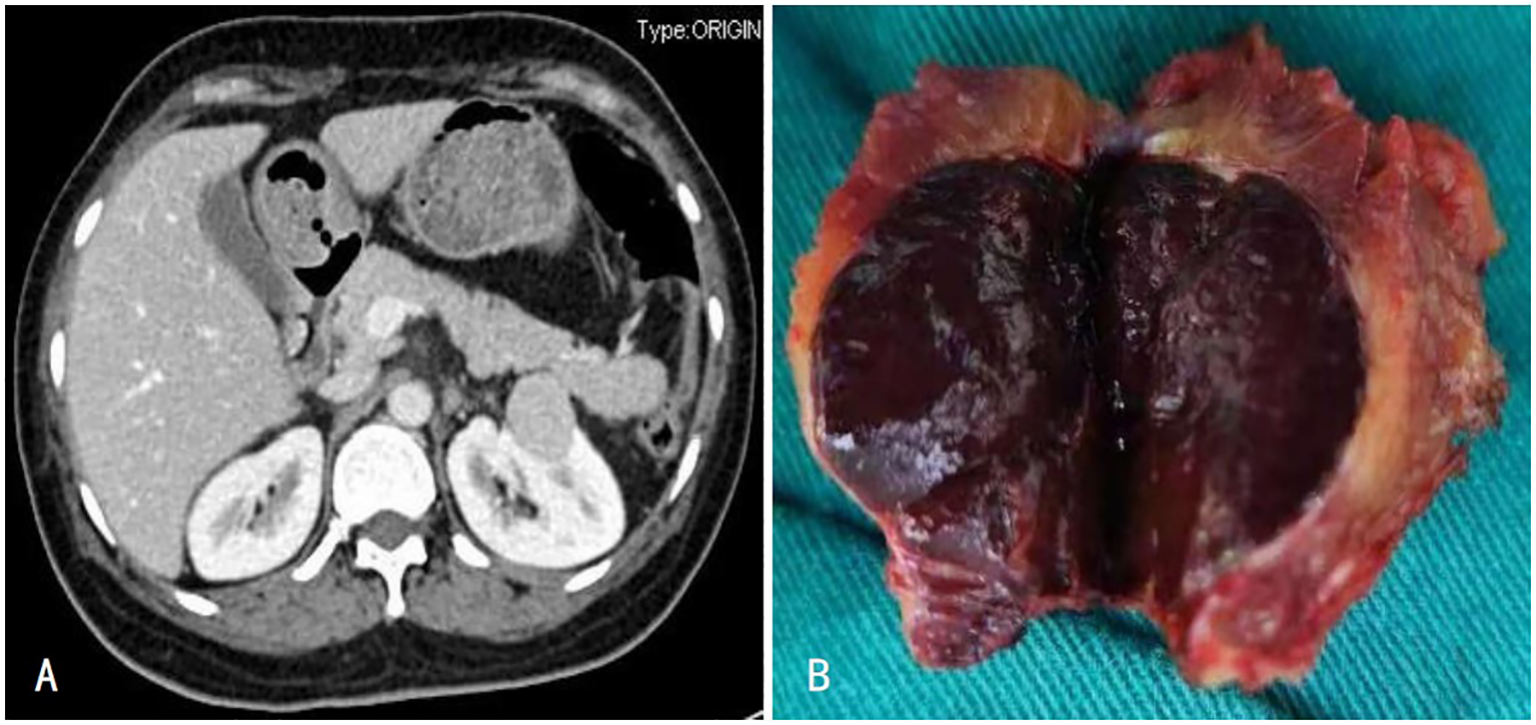
**(A)** Enhanced CT showed enhancement of the left renal tumor with capsule; **(B)** The left renal tumor was a dark red mass of tissue.

A 50-year-old woman underwent a computed tomography (CT) scan on October 25, 2023, which revealed a left renal mass. The lesion exhibited an abnormal enhancement, raising the suspicion of a neoplastic lesion located at the middle–upper pole of the left kidney, measuring approximately 3.1 × 2.6 cm (**Figure 1**). She reported no significant symptoms. She had experienced hypertension for the past 4 months, which was well controlled with oral medication. Additionally, she underwent open surgery for bilateral adrenal pheochromocytomas and splenectomy 18 years ago. After further evaluation, pheochromocytoma recurrence was ruled out, and laparoscopic left partial nephrectomy was performed. The postoperative pathology confirmed the presence of splenic tissue. She was followed up for 12 months, with stable disease and no new findings of splenic disease.

### Case 2

**Figure 2:**
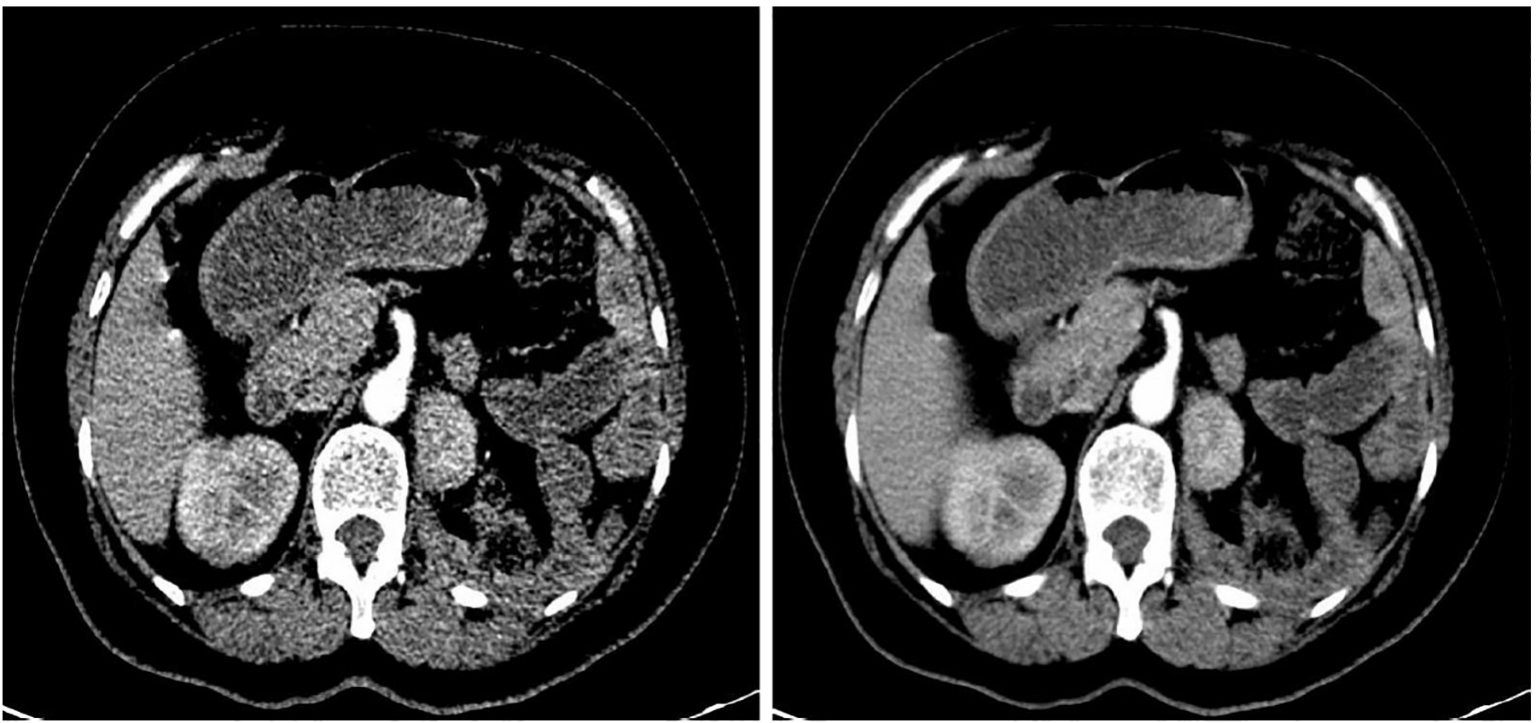
Contrast-enhanced CT revealed a tumor in the left renal fossa.

A 62-year-old woman underwent a CT scan on October 26, 2023; the findings revealed a homogeneously enhancing mass measuring approximately 3.6 × 2.8 cm (**Figure 2**) in the left renal fossa, which was suspected to be a neoplastic lesion. The patient reported no significant symptoms and had no underlying medical conditions. She had undergone left radical nephrectomy and splenectomy for a large renal tumor 13 years ago. Suspecting tumor recurrence, she underwent tumor resection of the left renal fossa under general anesthesia. The postoperative pathology confirmed the presence of splenic tissue. She developed an intestinal fistula postoperatively, which was successfully treated conservatively over 6 months. She was followed up for 12 months, with stable disease and no new findings of splenic disease.

## Discussion

The diagnosis of splenic tissue implantation or hematogenous metastasis to other parts of the body, often misdiagnosed as tumors in other locations, is referred to as splenic disease. It can manifest in any body cavity, except the spleen itself, including the abdominal cavity, retroperitoneum, and pancreas (4). Its association with urinary system diseases is relatively rare, as the latter primarily involve the kidneys, urinary tract, and surrounding tissues. The occurrence of ectopic splenic tissue in the kidney or renal fossa is termed renal-splenic disease. In clinical practice, differentiating renal-splenic disease from renal cancer or other benign renal tumors is difficult, often resulting in misdiagnosis as renal or recurrent tumor. This misdiagnosis can result in unnecessary surgical interventions.

Herein, we report two cases of renal-splenic disease that developed after surgical treatment. Furthermore, we provide a literature review based on a MEDLINE (PubMed) database search, identifying 37 cases of renal-splenic disease (**Table 1**). The earliest case was reported by Rao AK in 1976 (5), whereas the most recent one was published by Oyebola T in *BJU International* (6).

**Table 1:** Epidemiologic and clinical characteristics of 39 confirmed cases with concurrent renal and splenic pathologies.

No.	Year	Author	Age	Sex	Side	Location	Dimension	Symptoms	Diagnosis	Time of splenectomy	History of cancer
1	1976	Rao AK (5)	/	/	/	/	/	/	/	/	/
2	1988	Turk CO (13)	/	/	/	/	/	/	/	/	/
3	1991	Bock DB (14)	46	M	Left	Kidney	9	Abdominal discomfort	Surgery	27	No
4	1993	Forino M (3)	/	/	/	/	/	/	Biopsy	/	No
5	1994	Kearns CM (15)	34	M	Left	Kidney	4	Abdominal discomfort	Hybrid SPECT/CT	6	No
6	1994	Kearns CM (15)	66	F	Left	Kidney	10	Abdominal discomfort	Hybrid SPECT/CT	18	No
7	1994	Servadio Y (16)	33	M	Left	Kidney	6.2	Abdominal discomfort;Frequent micturition	Hybrid SPECT/CT	20	No
8	1996	Kiser JW (17)	48	M	Left	Kidney	6.0	Serendipity	Hybrid SPECT/CT	No time to record	No
9	2000	Sikov WM (18)	48	M	Left	Kidney	5	Serendipity	Surgery	41	No
10	2000	Mandosse P (19)	/	/	Left	Kidney	/	/	Surgery	/	/
11	2001	Pumberger W (20)	4	M	Left	Renal fossa	2	Serendipity	Hybrid SPECT/CT	2	Yes
12	2001	Echenique Elizondo M (21)	42	M	Left	Kidney	3	Abdominal discomfort	Surgery	No time to record	No
13	2003	Yuan S (11)	51	F	Left	Kidney	14.1	Abdominal discomfort	Surgery	No	No
14	2003	Berman AJ (22)	43	M	Left	Kidney	5.5	Serendipity	SPIO	17	No
15	2005	Dwyer NT (23)	/	/	/	/	/	/	/	/	/
16	2006	Page JB (24)	55	M	Right	Kidney	4	Serendipity	Surgery	No time to record	No
17	2007	Umemoto S (25)	55	M	Left	Renal fossa	5	Serendipity	Hybrid SPECT/CT	4	Yes
18	2007	Gürses B (26)	31	M	Left	Kidney	3	Serendipity	SPIO	20	No
19	2008	Onuki T (27)	65	M	Left	Renal fossa	2	Serendipity	Hybrid SPECT/CT	8	Yes
20	2008	Onuki T (27)	71	M	Left	Renal fossa	2	Serendipity	Hybrid SPECT/CT	9	Yes
21	2009	Al Ahmad A (9)	2.2(26个月)	F	Left	Kidney	4	Hematuria	Surgery	No	No
22	2009	Pérez Fentes D (28)	49	M	Left	Kidney	4.7	Abdominal discomfort	Hybrid SPECT/CT	27	No
23	2010	Brown JD (29)	22	M	Left	Kidney	3	Serendipity	Hybrid SPECT/CT	10	No
24	2011	Vercher-Conejero JL (30)	42	M	Left	Kidney	2	Abdominal discomfort	Hybrid SPECT/CT	31	No
25	2014	Cho SG (31)	40	M	Left	Kidney	5	Serendipity	Hybrid SPECT/CT	20	No
26	2015	Lamin E (32)	42	M	Left	Kidney	4.6	Abdominal discomfort	Biopsy	31	No
27	2017	Williamson SR (33).	66	M	Left	Kidney	5.8	Serendipity	Biopsy	No time to record	No
28	2017	Neufeld EA (34)	36	F	Left	Kidney	4	Serendipity	Hybrid SPECT/CT	30	No
29	2018	Tordjman M (7)	29	M	Left	Kidney	4	Serendipity	Surgery	No	No
30	2018	Jafari H (10)	80	F	Left	Kidney	4	Abdominal discomfort	Biopsy	No	No
31	2018	Tandon YK (4)	67	M	Left	Kidney	5.2	Abdominal discomfort	Biopsy	49	No
32	2018	Tandon YK (4)	65	M	Left	Renal fossa	3	Serendipity	CT	No time to record	No
33	2018	Tandon YK (4)	53	M	Left	Kidney	4	Serendipity	Surgery	No time to record	Yes
34	2019	McAlpine K (35)	58	F	Left	Kidney	3	Serendipity	Hybrid SPECT/CT	1	Yes
35	2019	Zugail AS (8)	29	M	Left	Kidney	4	Frequent micturition	Surgery	No	No
36	2022	Bray G (1)	55	F	Right	Kidney	2.5	Serendipity	Hybrid SPECT/CT	30	No
37	2024	Oyebola T (6)	22	M	Left	Kidney	10	Serendipity	Biopsy	No	/
38	2024	Present case	50	F	Left	Kidney	3	Serendipity	Surgery	18	Yes
39	2024	Present case	62	F	Left	Renal fossa	3.6	Serendipity	Surgery	13	Yes

Hybrid SPECT/C :99mTc-labelled heat-denatured erythrocytes and computed tomography.

SPIO, Superparamagnetic Iron Oxide-Enhanced MRI.

Among the 39 patients with renal-splenic disease, 25 and 9 were men and women, respectively, whereas the sex of the remaining 5 patients could not be determined from the literature. The disease occurred on the left side in 33 cases and on the right side in 2, whereas in 4 cases, the involved side was unknown. Furthermore, the disease affected the kidneys in 29 cases and renal fossa in 6, whereas in 4 cases, the location was unspecified.

In the 39 cases, the diagnosis was made using various imaging and diagnostic methods: 15 cases using 99mTc-labeled heat-denatured erythrocytes and CT; 2 using superparamagnetic iron oxide-enhanced magnetic resonance imaging (MRI); 1 using enhanced CT; 6 via biopsy, and 12 after surgical resection. In three cases, the diagnostic method was not specified. The majority of diagnoses were incidental (n = 21), with 11 cases discovered during investigations on abdominal discomfort, 2 cases with a symptom of frequent urination, 1 case with hematuria, and 5 cases with unspecified symptoms. The patients’ ages ranged from 2.2 to 80 years, with an average age of 45.91 years. Of the 28 patients with a history of splenectomy, the time from splenectomy to diagnosis ranged from 1 to 49 years, with an average of approximately 20 years. Imaging showed lesions measuring 2 to 14.1 cm, with an average diameter of 4.71 cm. Of the 39 patients, 8 had a history of malignant tumors whereas 26 had none.

The primary cause of splenic disease is the transfer of splenic tissue to the kidneys or renal fossa following spleen injury (7). Our analysis revealed that renal-splenic disease was more common in men (25:9), which could be attributed to the higher incidence of spleen injury in this population. Splenic tissue metastasis may occur through implantation or hematogenous spread, with the left kidney more frequently involved. This supports the hypothesis that splenic tissue implantation is the primary route of metastasis, consistent with current literature reports (8). Most patients with renal-splenic disease experience nonspecific symptoms and are incidentally diagnosed. However, when ectopic splenic tissue compresses surrounding structures, such as the renal pelvis or ureter, it can lead to urinary obstruction, hydronephrosis, or urinary tract infection, presenting as nonspecific abdominal discomfort, frequent urination, or hematuria (6, 9).

Renal-splenic disease affects all age groups, without specific age predilection. In our cohort, the youngest patient was 2.2 years old (9) and the oldest was 80 years old (10). Among the 28 patients with a history of splenectomy, the time from splenectomy to diagnosis ranged from 1 to 49 years, with an average of approximately 20 years. This long latency can reduce clinicians’ awareness of the need to screen for renal-splenic disease, thereby increasing the risk of misdiagnosis.

Interestingly, nearly half of the cases were diagnosed via imaging, such as Hybrid SPECT/CT (99mTc-labeled heat-denatured erythrocytes and computed tomography) and SPIO (Superparamagnetic Iron Oxide-Enhanced MRI) (1, 15–17, 20, 22, 25–31, 34, 35), and six were diagnosed via biopsy (3, 4, 6, 10, 32, 33). However, as many patients with renal-splenic disease have a history of abdominal surgery, which disrupts normal anatomical structures, performing a biopsy may be difficult. Furthermore, owing to the rich vascular supply of splenic tissue, biopsy may cause bleeding or injury to surrounding organs, although these complications were seldom reported in the literature.

There were 12 cases who were diagnosed after surgical resection, including partial nephrectomy, radical nephrectomy, or renal fossa tumor excision. Surgical resection has certain advantages, particularly in confirming the diagnosis and removal of the lesion. However, it is also associated with surgical complications, prolonged recovery, and potential functional impairment. Therefore, the decision to proceed with surgery should be based on the patient’s specific condition, disease characteristics, and overall health status. Although surgical complications were not reported in the literature, one patient in our cohort developed postoperative enterocutaneous fistula after a prior laparoscopic left nephrectomy.

In conclusion, for asymptomatic patients with renal-splenic disease, conservative treatment does not generally lead to severe disease progression. This is particularly true for patients with a history of splenectomy as the regenerated splenic tissue may perform functions similar to those of the original spleen, such as 1) participant in immune function, i.e., the removal of pathogens and aging red blood cells; 2) blood filtration, i.e., the removal of impurities and aging cells from the blood; 3) blood storage, i.e., the storage of red blood cells and platelets; and 4) hematopoiesis, i.e., the resumption of blood cell production under certain conditions (e.g., bone marrow failure) as they are ectopic splenic tissue (11). While ectopic splenic tissue is typically benign, it may grow large enough to compress adjacent structures, which may prompt surgical removal to prevent potential complications. Interestingly, previous reports have indicated that retroperitoneal ectopic splenic tissue rarely exceeds 4 cm in size (12). However, in our cohort, the average diameter of the lesions was 4.7 cm, which raises questions regarding whether conservative treatment is truly appropriate in these cases or if it delays the optimal time for surgery.

Additionally, we observed that approximately 70% of the patients had a history of splenectomy, with an average of 20 years between splenectomy and the onset of renal-splenic disease. This indicates that splenic tissue slowly generates and that conservative management or active monitoring could be reasonable options.

Although pathological examination remains the gold standard for diagnosis, Hybrid SPECT/CT (99mTc-labeled heat-denatured erythrocytes and computed tomography) and SPIO offer comparable diagnostic value, helping patients avoid invasive procedures or treatments (7).

In the reviewed cases where biopsy or surgical resection was performed, neither Hybrid SPECT/CT nor SPIO were employed, highlighting the diagnostic challenges of renal-splenic disease in the context of urinary system diseases. These challenges include preoperative diagnosis difficulties, increased surgical risks, postoperative complications, and potential impacts on the patient’s overall condition.

## Conclusion

Herein, we report 48 years of case data on renal-splenic disease and offer a comprehensive review to inform clinical practice. Correct identification of renal-splenic disease is crucial for patient safety and improve treatment outcomes. Providing rational diagnostic and therapeutic strategies is essential to enhance clinical practice, minimize recovery time and complications, and ultimately improving patients’ quality of life.

## Data Availability

The original contributions presented in the study are included in the article/supplementary material. Further inquiries can be directed to the corresponding authors.
